# miR-146a Overexpression in Oral Squamous Cell Carcinoma Potentiates Cancer Cell Migration and Invasion Possibly *via* Targeting HTT

**DOI:** 10.3389/fonc.2020.585976

**Published:** 2020-11-13

**Authors:** Liping Wang, Yunxin Chen, Yongyong Yan, Xueqi Guo, Ying Fang, Yucheng Su, Lijing Wang, Janak L. Pathak, Linhu Ge

**Affiliations:** ^1^ Affiliated Stomatology Hospital of Guangzhou Medical University, Guangzhou Key Laboratory of Basic and Applied Research of Oral Regenerative Medicine, Guangzhou, China; ^2^ Institute of Oral Disease, Guangzhou Medical University, Guangzhou, China; ^3^ Chinese Academy of Medical Sciences, Peking Union Medical College, Beijing, China; ^4^ School of Life Science and Biopharmaceutics, Vascular Biology Research Institute, Guangdong Pharmaceutical University, Guangzhou, China

**Keywords:** oral squamous cell carcinoma, mir-146a, Huntington gene, migration, invasion, metastasis, tumorigenesis

## Abstract

Huntingtin (HTT) is one of the target genes of miR-146-a and regulates various cancer cell activities. This study aims to explore the miR-146a expression pattern in oral squamous cell carcinoma (OSCC) and its role and mechanism in OSCC progression and metastasis *via* targeting the HTT gene. OSCC tissue and non-cancerous matched tissue (NCMT) were obtained from 14 patients. OSCC cell lines and normal HOK cells were used to analyze migration and invasion assay. OSCC-induced miR-146a knockout mice (B6.Cg-Mir146tm1.1Bal) model was developed. Transwell cell migration/invasion and scratch wound assays were used to investigate the OSCC cell migration and invasion *in vitro*. Kaplan-Meier survival analysis was used to investigate the association of HTT expression patterns in cancer tissue with patient survival percentage and duration. Pearson’s correlation analysis tested the association between miR-146a and HTT expression in OSCC tissues. miR-146a mimic and inhibitor transfection were performed to overexpress and knockdown the miR-146a in OSCC cells, respectively. miR-146a expression was highly upregulated in OSCC tissues and OSCC cell lines. Cancer cell migration/invasion was enhanced in miR-146a overexpressed cells and reduced in mi-R146a knockdowned cells. HTT expression was reduced in OSCC tissues and cell lines compared to NCMT and HOK cells, respectively. HTT expression was downregulated in miR-146a overexpressed OSCC cells and upregulated in miR-146a knockdowned OSCC cells. The expression pattern of miR-146a in OSCC cell lines and tissues was inversely correlated with HTT expression. Prediction of miRNA target analysis showed that HTT possesses the binding sites for miR-146a. HTT overexpression in OSCC tissues was associated with patients’ higher survival percentage and duration. HTT knockdown in OSCC cells enhanced miR-146a expression and cell migration/invasion. Inducing OSCC in miR-146a knockout mice increased the HTT expression in tongue tissue and alleviated the cancer aggressiveness and epithelial damage. Overexpressed miR-146a in OSCC targets the HTT gene and enhances cancer cell migration/invasion unraveling the possible role of HTT in miR146a-mediated OSCC cell migration and invasion.

## Introduction

Oral cavity cancer is the most common head and neck cancer with poor prognosis and a high recurrence rate (1). Oral squamous cell carcinoma (OSCC) accounts for more than 90% of all head and neck cancer and is the sixth most common cancer worldwide (2, 3). The incidence rate of OSCC is increasing rapidly, especially among young and middle-aged individuals resulted from smoking and alcohol abuse. Despite the progress of surgery, radiotherapy, and chemotherapy treatments, the 5-year survival rate of OSCC patients was still less than 50% in the last 30 years (2, 4). OSCC has a high rate of metastasis in the head and neck region due to local invasion and due to lack of early diagnostic markers (5). The pathogenesis of OSCC is a complicated process, and the molecular mechanisms of OSCC tumorigenesis, progression, and metastasis are still unclear. Therefore, the molecular level understanding in OSCC progression and metastasis is necessary to unveil novel diagnostic and therapeutic targets.

MicroRNAs (miRNAs) are an evolutionarily conserved group of small single strand non-coding RNA molecules of 20 to 22 nucleotides that regulates mRNA expression at transcriptional and post-transcription level (6, 7). Emerging pieces of evidence had indicated that the mature miRNAs play critical roles in a broad range of physiologic and pathologic processes, such as development, cell proliferation, differentiation, apoptosis, signal transduction, and development of diseases, including inflammation and cancers (8–13). Literature had reported the involvement of various miRNAs, including miR-146a, miR-145, miR-433, miR-195-5p, and miR-375 in OSCC (14–17). Overexpression or underexpression of miRNAs regulates the OSCC development and progression. The role of miR-146a in various cancer etiology, including blood, breast, cervical, kidney, liver, and lung cancer, had been extensively studied (18). Only pieces of literature are available regarding the role of miR-146a in OSCC, and the results are controversial (17, 19, 20). Hung and colleagues reported the upregulation of miR-146a in OSCC tissues and blood circulation of OSCC patients, and the involvement in OSCC oncogenicity (17). Similarly, Min and colleagues reported a higher expression of miR-146a in OSCC (19). In contrast, Shi and colleagues reported the loss of miR-146a expression in high-grade oral cancer tumors and re-expression of miR-146a reduced oncogenic phenotypes and metastasis (20). Therefore, further studies are necessary to elucidate the exact expression pattern of miR-146a in OSCC as well as its role and mechanism in OSCC progression.

miR-146a targets specific genes, regulates signaling pathways, and involves in cancer biology (18). miR-146a targets IRAK1 and TRAF6 to promote NF-κB signaling in oral, cervical, breast, and prostate cancer (17, 18, 21). Similarly, miR-146a targets EGFR to promote growth and proliferative signaling of various cancer cells, including breast, liver, lung, prostate, and gastric cancer (18). However, other target genes of miR-146a and their role in OSCC etiology have not been fully understood. HTT gene mutation causes a neurodegenerative Huntington’s disease (22). Most of the HTT-related studies are mainly focused on the role of HTT in the nervous system. The expression of the HTT gene and protein is ubiquitous. Some recent studies have reported the role of HTT in cancer development and progression. HTT expression is downregulated in breast cancer and regulates cancer cell differentiation *via* the maintenance of tight junctions (23). HTT gene expression is downregulated in oral cancer tissues and is one of the candidate genes involved in oral cancer biology (24). Moreover, miR-125b, miR-146a, miR-150, and miR-214 target both human and mice HTT and regulate diverse cellular processes (25, 26). However, whether the miR-146a targets the HTT gene in OSCC to regulate tumorigenesis and metastasis is still unknown.

In this study, we hypothesized that miR-146a targets the HTT gene to regulate OSCC cell migration and invasion. We found overexpressed miR-146a and underexpressed HTT in OSCC clinical tissues and cell lines. The overexpressed miR-146a downregulated HTT expression in OSCC cells, suggesting the HTT targeting potential of miR-146a. Overexpressed miR-146a or underexpressed HTT enhanced OSCC cell migration/invasion. Inducing OSCC in miR-146a knockout mice mitigated the cancer aggressiveness and tongue epithelial damage compared to in wild-type mice. For the first time, our study reported the overexpression of miR-146a in OSCC as an inducer of cancer cell migration and invasion possibly *via* targeting the HTT gene.

## Materials and Methods

### OSCC Tissue Collection

A total of 14 patients with OSCC were included in this study. The independent diagnosis of each case was confirmed by both pathologists and physicians following the standard criteria (27). Surgical resection of primary tumors along with paired non-cancerous matched tissues (NCMT) from OSCC patients was collected with the written informed consent from the patients. NCMT sample was obtained from 2 cm distance of the tumor tissue. Tissue specimens were stained with H&E staining to distinguish cancerous tissue from NCMT. This study was approved by the Ethical Institutional Review Board of Affiliated Stomatology Hospital of Guangzhou Medical University (ethical approval number: KY2017018). All the procedures were per institutional ethical standards. All samples were obtained during tumor removal surgery and were frozen immediately in liquid nitrogen and stored at −80°C until the detection of miR-146a and HTT. The tumors underwent TNM classification, according to the American Joint Committee on Cancer (AJCC) system (27). The patient’s characteristics and demographics are presented in **Table 1**.

**Table 1:** Clinical parameters of OSCC patients.

Demographics	Patient number (n = 14)
Gender	
Male	7
Female	7
Age	
<50	6
>50	8
Lymph node metastasis	
Negative	3
Positive	11
TNM stage	
II	3
III	11

### *In Vitro* Cell Culture Study

SCC9, SCC25, and CAL27 human oral squamous carcinoma cell lines were used in this study. SCC25 (CRL-1628) was purchased from ATCC. HOK cell line was obtained from ScienCell (Chemie Brunschwig, Basel, CH). SCC9 and CAL27 were obtained from the Key Laboratory of Oral Medicine, Affiliated Stomatology Hospital of Guangzhou Medical University. SCC25 cells were cultured in DMEM/F12 supplemented with 400 ng/ml hydrocortisone and 10% fetal bovine serum. SCC9 and CAL27 cells were cultured in DMEM/F12 supplemented with 10% FBS and 1% Penicillin-Streptomycin (Gibco, 15140122). All cells were incubated in an atmosphere of 5% CO_2_ and saturated moisture at 37°C.

### Cell Transfections

miR-146a mimic (cat. #4464066), miR-146a mimic negative control (designated miR-146a mimic NC, cat. #4464058), miR-146a inhibitor (cat. #4464084), and miR-146a inhibitor negative control (designated miR-146a inhibitor NC, cat. #4464076) were obtained from Thermo Fisher (life technology, Carlsbad, CA). Transfection was performed using GenMute @ (SignaGen) according to the manufacturer’s protocol. Briefly, cells were seeded in 6-well plates at 30%–40% confluence for 24 h, transfected with miR-146a mimic, miR-146a mimic negative control, miR-146a inhibitor, and miR-146a inhibitor negative control at a final 50 nM concentration for 48 h.

### Knockdown of HTT Gene in OSCC Cells

HTT gene in OSCC cells was knock downed using si-RNA. HTT knockdown efficiency of three different si-RNAs (si-HTT1, si-HTT2, si-HTT3) in SCC9 and CAL27 cells was tested. Since, si-HTT1 (targeted sequence: GCACCTTCCTCCTGAGAAA, Guangzhou RIBOBIO) showed the highest inhibition of HTT expression, we used the si-HTT1 to downregulate the HTT in OSCC cells. HTT knockdown efficiency, miR-146 expression pattern, and OSCC cell migration and invasion were further analyzed.

The cells in the logarithmic growth phase were seeded in a 6-well plate. When the cell fusion degree was 80%, siRNA transfection was performed. The specific steps were performed according to the method provided in the instructions of the siRNA transfection kit. After the cells were transfected for 48 h. The morphological changes of cells were observed under an inverted light microscope, and total RNA was extracted, and the silencing effect was detected by quantitative real-time PCR (qRT-PCR).

### Quantitative Real-Time PCR Assay

Total RNA was extracted from cultured cells, OSCC clinical tissues, and mice tongue tissues using TRIzol Reagent (Invitrogen, CA, USA). The miRNA was extracted from cultured cells using miRNAiso for small RNA reagent (TaKaRa, Dalian, China). The RNA samples were then reverse-transcribed into cDNA with the PrimeScript RT Master Mix (TaKaRa, Dalian, China) and the SYBR PrimeScript miRNA RT-PCR Kit (TaKaRa, Dalian, China). Real-time PCR was performed with the SYBR Premix Ex Taq II and the SYBR PrimeScript miRNA RT-PCR Kit (TaKaRa, Dalian, China), using an Applied Biosystems 7500 Sequence Detection System (Applied Biosystems, Foster City, CA, USA). The expression of HTT mRNA was quantified with GAPDH mRNA expression as an endogenous control. The levels of miR-146a were quantified with U6 control. Quantification of the relative levels was determined by the ^ΔΔ^Ct method (28). Primers used for qPCR are listed in **Table 2**.

**Table 2:** Primers used in this study.

Gene	Forward Primer	Reverse Primer
GAPDH	GGAGCGAGATCCCTCCAAAAT	GGCTGTTGTCATACTTCTCATGG
Gapdh (M)	AGGTCGGTGTGAACGGATTTG	GGGGTCGTTGATGGCAACA
U6	GGAACGATACAGAGATTAGC	TGGAACGCTTCACGAATTTGCG
HTT (H)	GGGTGGGAAGAAGTCGTCTAG	GCTATGGAGCGGGTATCTGTT
miR-146a	UGAGAACUGAAUUCCAUGGGUU	ACACTCCAGCTGGGTGGTGCGGAGAGGGCCC
Htt (M)	CTGATGAAGGCTTTCGAGTCG	TGATTCACACGGTCTTTCTTGG

### Cell Invasion Assay

Cell invasion assay was performed using Transwell chambers (8.0 μm pore size; Corning, US). The chambers were prepared by thawing Matrigel (BD Bioscience, San Jose, CA) at 4°C, and then 100 μl of the thawed Matrigel was added to each insert. After incubating at room temperature for 1 h, the unsolidified liquid was gently removed by pipette. Transfected cells were starved overnight and then seeded in the upper chamber at a density of 2.5 × 10^6^ cells/ml in 200 µl of medium with no FBS. A medium with 10% FBS (600 µl) was added to the lower chamber. Following a 24 h-incubation at 37°C with 5% CO_2_, non-invading cells in the upper chamber were removed with a cotton swab, and invading cells were fixed in 4% paraformaldehyde and stained with 0.5% crystal violet. Photographs were taken randomly from five fields of each membrane. The number of invading cells was expressed as the average number of cells per microscopic field over five fields.

### Cell Migration Assay

For migration assays, a protocol similar to the invasion experiment. However, the upper chamber was not pre-coated with Matrigel, and cells in the upper chamber were seeded at a density of 1.0 × 10^6^ cells/ml.

### Scratch Wound Assay

The cell migration was measured by scratch wound assay. Transfected OSCC cells were cultured in 6-well plates to 100% confluence as monolayers and then scratched with a 200-µl sterile pipette tip (29). The medium and cell debris were aspirated away by PBS and replaced with 2 ml of fresh serum-free medium. The area between wound gaps was measured at different time points under an inverted phase-contrast microscope (Olympus, Germany) and then calculated by ImageJ software. Five randomly selected fields along each wound were marked, and each experiment was conducted in triplicate.

### Mouse Model for Oral Cancer

Male C57BL/6 mice (wild-type) 6–7 weeks old, weighing 250–350 g, were obtained from Medical Laboratory Animal Center of Guangdong Province. miR-146a knockout mice (B6.Cg-Mir146^tm1.1Bal^) were obtained from the Jackson Laboratory. All mice were housed at the Specific Pathogen Free (SPF) animal facility at Guangzhou Medical University according to animal protocol and regulation of the University Laboratory Animal Resources. All animal experiments were approved by Guangzhou Medical University Institutional Animal Care and Institutional Biosafety Committee (ethical approval number: 2020-002). Animal experiments were performed following the ARRIVE guidelines. OSCC was induced in wild-type (9 mice) and miR-146a (9 mice). The OSCC in mice was induced following the protocol established by our lab (30), as illustrated in **Supplementary Figure 1**. In brief, the mice were treated with freshly prepared 4-NQO (Cat# N8141; Sigma, St Louis, MO) for 10 weeks and then treated with normal drinking water for 10 weeks. 4-NQO (Sigma Chemical) was dissolved in propylene glycol (final concentration 5 mg/ml) and then added to the drinking water at 60 μg/ml, keeping at −20°C. Drinking water containing NQO was freshly prepared every week in deionized water and was stored in the dark at 4°C until used. Bottles containing 4-NQO-supplemented water were wrapped with foil to preclude photodegradation of the carcinogen and were changed at two- to three-day intervals throughout the study. The drinking water was changed every day, and mice were allowed access to the drinking water at all times while receiving treatment. The mice were anesthetized (xylazine, intraperitoneal injection, 0.13 mg/kg body weight) and euthanized by exsanguination prior to tissue collection. Mice were euthanized at 0, 16, and 20 weeks after treatment, respectively. The tongue tissues were collected and stored at −80°C for the histology and qPCR.

### Hematoxylin and Eosin Staining

The tongue tissues were fixed in 4% paraformaldehyde for 16 hours, then dehydrated and embedded in paraffin. Sections (3 μm thick) were stained with hematoxylin and eosin (H&E). The sections of the tongue epithelium were photographed with an inverted optical microscope.

### Immunochemistry

Immunohistochemistry was performed to observe the expression of HTT. The anti-HTT antibody (ab109115, Abcam) was incubated overnight at 4°C, followed by incubation with biotinylated anti-rabbit secondary antibodies for one hour and counterstained with hematoxylin to detect HTT expression on human OSCC tissue sections. The number of HTT-positive cells and nucleated cells in five random fields of view on each human OSCC tissue and NCMT tissue section were counted Using ImageJ software. Their ratio (HTT-positive cells/nucleated cells) was used for statistical analysis. **Table 1** summarizes the clinicopathological characteristics of patients with OSCC.

### Kaplan-Meier Survival Analysis and Pearson’s Correlation Analysis

Kaplan-Meier survival curve for 397 OSCC/HNSCC patients with high expression of HTT and 99 patients with low HTT expression was plotted to evaluate the survival percentage and duration as described previously (31). Primary data (survival duration and HTT expression) of the patients were taken from the Kaplan Meier plotter (http://www.oncolnc.org/). Pearson’s correlation analysis was performed using GraphPad Prism 7.04 software to investigate the correlation between miR-146a and HTT expression in OSCC tissues.

### Statistical Analysis

All statistical analyses were performed using GraphPad Prism 7.04 software and the SPSS 20.0 statistical package (SPSS, Inc., Chicago, IL, USA). Data were presented as the mean ± SD. A nonparametric unpaired Mann-Whitney test was used to compare the mean of two independent groups. Differences were examined using a one way ANOVA followed by Tukey-Kramer post-hoc test and independent samples t-test. Chi-Square was used to test differences between two or more group samples. P < 0.05 was considered statistically significant.

## Results

### miR-146a Is Highly Expressed in OSCC

**Figure 1:**
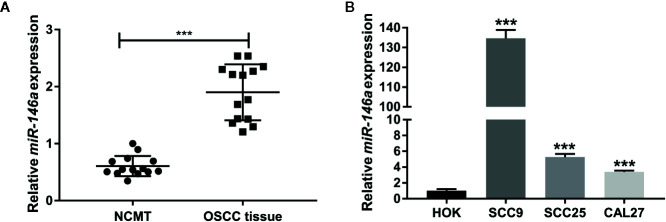
MiR-146a expression was upregulated in OSCC tissues and OSCC cell lines. **(A)** MiR-146a expression pattern in OSCC tissues and non-cancerous matched tissues (n = 14). **(B)** MiR-146a expression pattern in HOK cells and OSCC cell lines (n = 3). Data are presented as mean ± SD. The significant difference, ***P < 0.001 [in **(B)** compared to HOK-group]. NCMT, non-cancerous matched tissue; OSCC, oral squamous cell carcinoma.

To explore the role of miR-146a in OSCC, we analyzed the expression of miR-146a in OSCC tissues and NCMT from 14 patients. Patient characteristics, demographics, and clinical data are provided in **Table 1**. miR-146a was highly expressed in OSCC tissue compared to NCMT (**Figure 1A**). We also analyzed the miR-146a expression in OSCC cell lines and HOK cells. miR-146a expression was highly upregulated in all the OSCC cell lines tested (SCC9, SCC25, and CAL27) compared to HOK cells (**Figure 1B**). SCC9 showed the highest expression of miR-146 (135-fold higher) compared to HOK cells. The results of miR-146a expression from clinical samples directly correlated with the results from OSCC cell lines.

### Overexpression of miR-146a Promotes OSCC Cell Migration and Invasion

**Figure 2:**
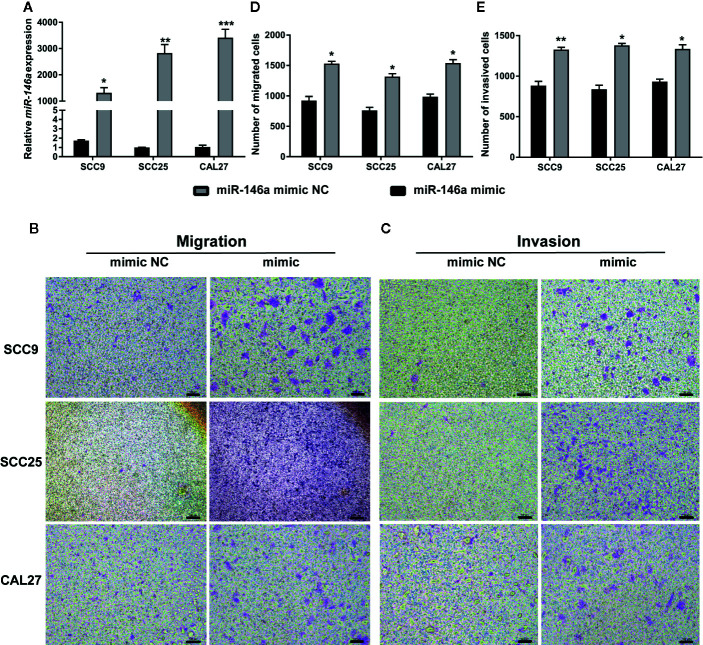
Transfection of miR-146a in OSCC cells enhanced cell migration and invasion. **(A)** miR-146a expression pattern in OSCC cells transfected with miR-146a negative mimic control or miR-146a mimic. Representative microscopic images of **(B)** OSCC cell migration, and **(C)** OSCC cell invasion assays. Purple dots and patches indicate the migrated and invaded cells. Quantitative analysis of **(D)** migrated, and **(E)** invaded cells from figure B and C. Data are presented as mean ± SD form three independent experiments in triplicate. Significant effect of miR-146a transfection, *P < 0.05, **P < 0.01, and ***P < 0.001. NC, negative control. Scale bar, 100 μm.

miR-146a was transfected in OSCC cells to analyze the effect of miR146a on OSCC cell migration and invasion. Transfection of miR-146a successfully upregulated the expression of miR-146a in SCC9, SCC25, and CAL27 cells (**Figure 2A**). In vitro cell migration and invasion assay, showed a higher number of migrated and invaded cells in miR-146a transfected SCC9, SCC25, and CAL27 cells (**Figures 2B, C**). Quantitative analysis data showed that overexpression of miR-146a enhanced the cell migration and invasion by ~1.5-fold in SCC9, SCC25, and CAL27 cells (**Figures 2D, E**). This result indicates that the overexpressed miR-146a in OSCC cells promotes cancer cell migration and invasion.

### Knockdown of miR-146a in OSCC Inhibits OSCC Cell Migration

**Figure 3:**
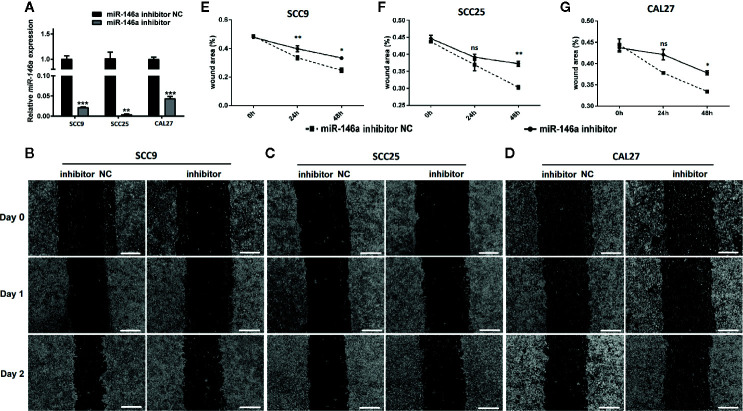
The knockdown of miR-146a inhibited the OSCC cell migration in scratch wound assay. **(A)** Relative expression of miR-146a in OSCC cells transfected with miR-146a inhibitor or miR-146a negative control. Representative microscopic images scratch wound assay in **(B)** SCC9, **(C)** SCC25, and **(D)** CAL27 cells. In the scratch wound assay, quantitative analysis was performed on migrating cells at 0, 24, and 48, **(E)** SCC9, **(F)** SCC25, and **(G)** CAL27 cells. Data are presented as mean ± SD form three independent experiments in triplicate. Significant effect of the miR-146a inhibitor treatment, *P < 0.05, **P < 0.01, and ***P < 0.001. NC, negative control. Scale bar, 100 μm.

We knockdowned miR-146a in OSCC cells to further confirm the role of miR-146a in OSCC migration and invasion. The transfection of miR-146a inhibitor dramatically reduced the expression of miR-146a expression in SCC9, SCC25, and CAL27 cells (**Figure 3A**). The scratch wound assay revealed that miR-146a knockdown reduces the migration of SCC9, SCC25, and CAL27 cells, as indicated by the higher wound area in the miR-146a inhibitor group (**Figures 3B–D**). The quantitative analysis of wound closure showed a higher wound area percentage in miR-146a inhibitor transfected SCC9, SCC25, and CAL27 cells (**Figures 3E–G**). Moreover, the knockdown of miR-146a inhibited the OSCC cell invasion (**Supplementary Figure 2**). Our results indicate that miR-146a overexpression and knockdown in OSCC have a stimulatory and inhibitory effect on cancer cell migration/invasion, respectively (**Figures 2** and **3**).

### HTT Expression Is Downregulated in OSCC

**Figure 4:**
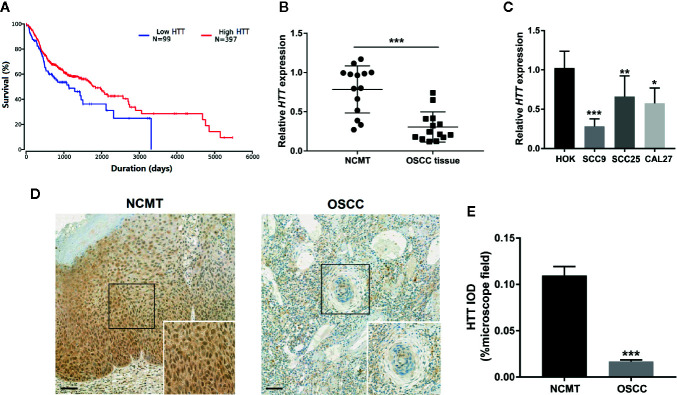
HTT expression pattern in OSCC correlated with survival rate and duration. **(A)** Kaplan-Meier survival curves for 397 OSCC patients with high HTT expression and 99 OSCC patients with low HTT expression. **(B)** HTT mRNA expression pattern in OSCC tissue and non-cancerous matched tissue (n = 14). **(C)** HTT mRNA expression pattern in HOK cells and OSCC cell lines (n = 3). **(D)** Representative images of immunohistochemistry sections showing HTT protein expression in OSCC tissue and non-cancerous matched tissue. **(E)** Quantitative analysis of HTT protein expression in immunohistochemistry sections (n = 14). Data are presented as mean ± SD. Significant difference, *P < 0.05, **P < 0.01, and ***P < 0.001. Scale bar, 100 μm.

Since miR-146a targets the HTT gene, we evaluated the expression pattern of the HTT gene in OSCC tissue and the role of the level of HTT expression on survival percentage and duration in oral cancer patients. We plotted the Kaplan-Meier survival curve from 397 OSCC/HNSCC patients with high HTT expression and 99 OSCC/HNSCC patients with low HTT expression. Cancer patients with higher HTT expression showed a better survival percentage with longer survival duration compared to the OSCC patients with HTT expression (**Figure 4A**). We also analyzed the HTT gene expression in OSCC tissues and NCMT of 14 patients as well as in OSCC cell lines. HTT expression was significantly downregulated in OSCC tissues compared to NCMT (**Figure 4B**). Similarly, the lower expression of the HTT gene was observed in OSCC cells (SCC9, SCC24, and CAL27) compared to HOK cells (**Figure 4C**). The immunohistochemistry study showed a lower expression of HTT protein in OSCC tissues compared to NCMT (**Figures 4D, E**). This result indicated that the downregulation of HTT in OSCC correlates with lower survival percentage and duration.

### miR-146a Expression Inversely Correlates With HTT Expression in OSCC

**Figure 5:**
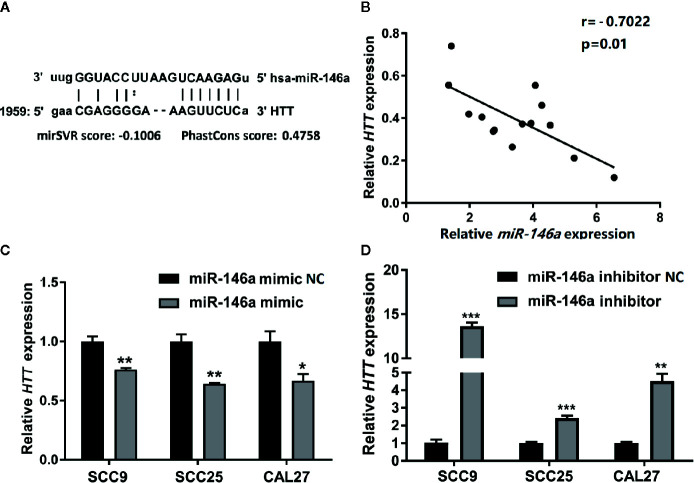
HTT expression was inversely related to miR-146a expression in OSCC tissues. **(A)** The binding site of miR-146a in the HTT gene. **(B)** Pearson analysis showing the correlation between miR-146a and HTT mRNA expression level in OSCC tissues (n = 14; r = −0.7022, P < 0.01). HTT expression pattern in **(C)** miR-146a transfected, and **(D)** miR-146a knockdowned OSCC cells. Data are presented as mean ± SD form three independent experiments in triplicate. Significant effect of treatment, *P < 0.05, **P < 0.01, and ***P < 0.001. NC, negative control. Scale bar, 100 μm.

Prediction of miRNA targets using TargetScanHuman online tool (http://www.targetscan.org/vert_72/) showed the binding site of miR146a in HTT gene (**Figure 5A**). We performed Pearson’s correlation analysis to investigate the correlation between miR-146a and HTT expression in OSCC. As expected, the miR-146a expression in OSCC tissues was inversely correlated (r = −0.7022, P = 0.01) with the HTT expression (**Figure 5B**). The miR-146a transfected OSCC cells showed a lower expression of the HTT gene (**Figure 5C**). The knockdown of miR-146a in OSCC cells robustly enhanced the HTT gene expression (**Figure 5D**).

### Knockdown of HTT Enhances OSCC Cell Migration and Invasion

**Figure 6:**
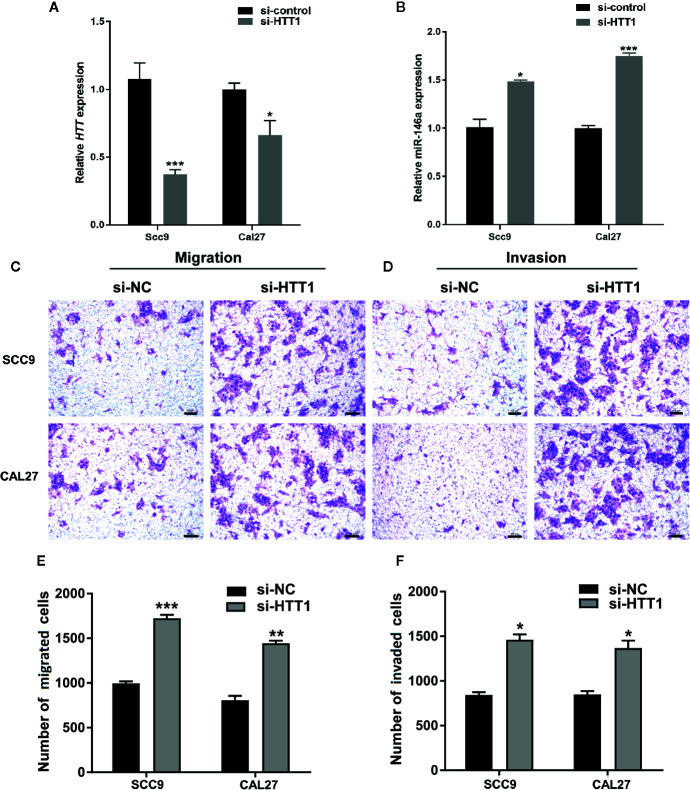
HTT inhibits the migration and invasion of OSCC cell lines. **(A)** HTT expression pattern in OSCC cell lines transfected with HTT si-RNA. **(B)** miR-146a expression pattern in OSCC cells transfected with HTT si-RNA. Representative microscopic images of **(C)** OSCC cell migration, and **(D)** OSCC cell invasion assays. Quantitative analysis of **(E)** migrated, and **(F)** invaded cells (**F**). Data are presented as mean ± SD form three independent experiments in triplicate. Significant effect of treatment compared to a si-NC group, *P < 0.05, **P < 0.01, and ***P < 0.001. NC, negative control.

To analyze the role of HTT in OSCC cell migration and invasion, we knockdowned the HTT gene by using HTT specific si-RNA (**Figure 6A**). Interestingly, knockdown of HTT upregulated the miR-146a expression in OSCC cells (**Figure 6B**). This result further confirms the inverse correlation between miR-146a and HTT expression in OSCC cells, as observed in **Figure 5**.

Cell migration and invasion assay showed a higher number of migrated and invaded OSCC cells in HTT knockdown groups (**Figures 6C, D**). The quantitative analysis showed ≥ 1.5-fold higher number of migrated and invaded cells in HTT knockdowned OSCC cells (**Figures 6E, F**). This result indicates the inhibitory effect of HTT on OSCC cell migration and invasion.

### Knockout of miR-146a Inhibits the OSCC Progression in Mice

**Figure 7:**
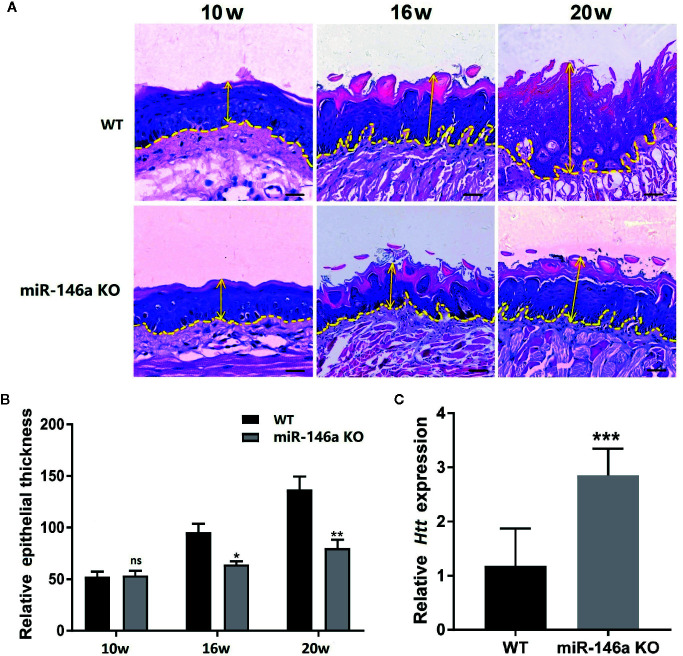
Knocking out of miR-146a in mice mitigates OSCC progression and tongue epithelial layer damage. **(A)** Representative images of histological section (H&E staining) of tongue tissue of wild-type and miR-146a knockout mice with OSCC. **(B)** Quantitative analysis of tongue epithelium thickness. **(C)** HTT mRNA expression pattern in wild-type and miR-146 knockout mice. Data are presented as mean ± SD form three independent experiments in triplicate. Significant effect of treatment compared to WT, *P < 0.05, **P < 0.01, and ***P < 0.001. KO, knockout; WT, wild-type; ns, not significant. Scale bar, 100 μm.

We tested the role of miR-146a in OSCC progression in miR-146a knockout mice with OSCC. Inducing OSCC in wild-type mice showed the tongue epithelial damage at week 10. The higher degree of carcinoma aggressiveness and epithelium damage was observed at week 16 and 20 in wild-type mice (**Figure 7A**). In miR-146a knockout mice, OSCC progression was relatively slow, and the epithelial damage was mitigated compared to in wild-type mice at week 16 and 20, respectively (**Figure 7A**). Tongue epithelial damage was increased in OSCC wild-type mice at week 16 and 20. Interestingly, OSCC-induced epithelial damage was alleviated in miR-146a knockout mice (**Figure 7B**). We also analyzed the HTT mRNA expression in the tongue tissue of OSCC mice at week 20 (**Figure 7C**). HTT mRNA expression was highly upregulated in the OSCC tongue tissue of miR-146a knockout mice compared to wild-type mice. This result indicates the protective role of the HTT gene against OSCC by reducing the cancer aggressiveness and tongue epithelial damage.

## Discussion

**Figure 8:**
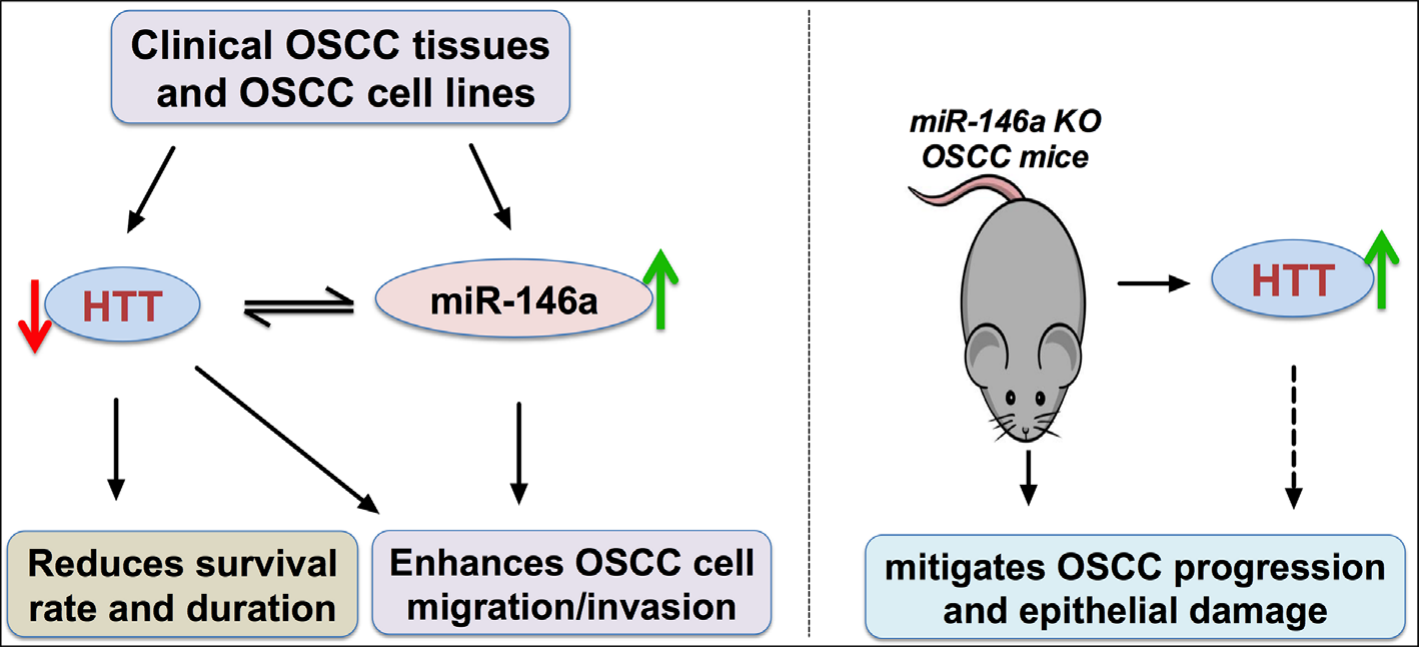
Scheme of miR-146a/HTT axis-mediated possible mechanism of OSCC cell migration and invasion. Red arrow, downregulation; green arrow, upregulation.

Evidence from literature demonstrated that the over or under expression of miR-146a in specific cancer acts as either a cancer suppressor or inducer by targeting specific genes (18). However, the expression pattern and function of miR-146a in OSCC is still a controversy (17, 19, 20). This study investigated: (a) the expression pattern of miR-146a in OSCC tissues and cells, (b) unravel the possible target gene of miR-146a, and (c) the role of miR-146a in OSCC development and metastasis. miR-146a was overexpressed, and its possible target HTT gene and protein was underexpressed in OSCC tissues and cell lines. The expression of miR-146a and the HTT gene in OSCC was inversely correlated. The higher expression of HTT in cancer tissues correlated with higher survival percentage and duration. Overexpressed miR-146a enhanced OSCC cell migration and invasion possibly by targeting the HTT gene. Inducing OSCC in miR-146a knockout mice enhanced HTT expression in tongue OSCC tissue and alleviated cancer aggressiveness and tongue epithelial damage. **Figure 8** illustrated the main findings of this study. Our results showed that the overexpressed miR-146a in OSCC possibly targets the HTT gene to induce cancer cell migration and invasion, suggesting the role of miR-146a and HTT in OSCC progression, metastasis, and invasion.

Overexpression of miR-146a had been reported in various cancers, including breast (8, 32), gastric (33), cervical (21), and bladder cancer (34). The expression pattern of miR-146a in OSCC is still a controversy as two studies reported overexpression (17, 19), and one study reported underexpression (20). In this study, OSCC tissues from 14 patients and OSCC cell lines SCC9, SCC25, and CAL19 showed a higher expression of miR-146a. Overexpressed miR-146a in various cancers such as breast (8), bladder (34), gastric (35), and prostate cancer (36) had been reported to regulate cancer cell migration, invasion, and metastasis. Similar to other types of tumors, the metastasis of OSCC mostly involves local invasion restricted to the head and neck region that frequently remain undetected until the advanced stage. Irani S had summarized the distant metastasis of OSCC, including heart, lung, bone, and brain (37). Therefore, it is crucial to unravel the role of miR-146a on OSCC metastasis and its mechanism. We overexpressed and knockdowned the miR-146a in OSCC cell lines to investigate the role of miR-146a in OSCC migration and invasion. miR-146a overexpression promoted and knockdown inhibited the OSCC cell migration and invasion *in vitro* (**Figures 2** and **3**). Our data indicated the possible role of overexpressed miR-146a on OSCC metastasis.

Differentially expressed miR-146a targets specific genes, affects gene transcription and translation, and regulates tumor biology (18). TRAF6, IRAK1, and SOX2 had been reported as a target gene of miR-146a in OSCC (17, 19, 20). miR-146a has specificity to target the HTT gene, and HTT plays a vital role in oral and breast cancer biology and many other cellular activities (23–26). This study hypothesized that the miR-146a might target the HTT gene to regulate OSCC progression and metastasis. We extensively analyzed the expression pattern of HTT and its role in OSCC (**Figure 4**). HTT gene and protein expression were downregulated in OSCC tissues with higher pathological grade. A similar result in OSCC expression was observed in SCC9, SCC25, and CAL27 cells (**Figure 5**). Our results suggest HTT as a possible diagnostic or therapeutic target in OSCC. Thion and colleagues had reported the anti-metastatic role of the HTT gene in breast cancer (23, 38). We unraveled the strong association between the lower expression of HTT in OSCC/HNSCC with reduced survival rate/duration and vice versa, indicating a critical role of HTT in OSCC.

Since high throughput sequencing analysis revealed the HTT gene as a putative target of miR-146a, we further analyzed the regulatory role of miR-146a on HTT gene expression in OSCC. The expression patterns of miR-146a and HTT in OSCC tissues were inversely correlated. These findings inmiR-146a overexpression downregulated, and knockdown upregulated the HTT gene/protein expression in OSCC cells. Similarly, knockdown of the HTT gene upregulated miR-146a expression in OSCC cells. Our results indicate the HTT targeting potential of miR-146a in OSCC.

Results from literature and our current study showed that the lower expression of HTT in oral and breast cancer is related to cancer metastasis and lower patient survival (23, 24, 38). In this study, we found that knockdown of the HTT gene in OSCC cells robustly enhanced the cancer cell migration and invasion *in vitro*. HTT gene expression was upregulated in the OSCC tongue tissue of miR-146a knockout mice. Aggressive OSCC in tongue tissue with a disrupted tongue epithelial layer was observed in wild-type mice. In contrast, the reduced aggressiveness of OSCC in tongue tissue and intact tongue epithelial layer was observed in miR-14a knockout mice. Our results indicate the role of overexpressed miR-146a on OSCC aggressiveness *via* targeting the HTT gene.

In this study, we evaluated the expression pattern of miR-146a and HTT gene/protein, as well as HTT targeting potential of miR-146a in well-characterized OSCC clinical samples, mouse model, and cell lines. We also investigated the role of miR-146a in OSCC cell migration, invasion, and cancer aggressiveness. Use of OSCC mice model in miR-146a knockout mice to investigate the role of miR-146a in OSCC and HTT expression in another novelty of this study. This is the first study to illustrate the expression pattern of HTT in OSCC tissues and cell lines, as well as the role of miR-146a in OSCC development and HTT expression in knockout mice model. Using only 14 OSCC patients is a limitation of this study. Another limitation of this study is that we did not investigate the molecular mechanism of miR-146a/HTT-mediated OSCC cell migration/invasion and metastasis. Therefore, future research recruiting more OSCC patients and unraveling the molecular mechanism of miR-146a/HTT-mediated signaling pathways in OSCC metastasis are strongly recommended. Knockdown of HTT in OSCC cells enhanced the cancer cells migration and invasion, and miR-146a knockout OSCC mice showed higher expression of HTT and inhibited the cancer aggressiveness and epithelial damage (**Figures 7A–C**). Based on these results we predict that miR-146a overexpression in oral squamous cell carcinoma potentiates cancer cell migration and invasion possibly *via* targeting HTT. However, the future *in vitro* and *in vivo* studies, overexpressing HTT in miR-146a overexpressed OSCC cells or mice model, as well as inhibiting HTT in miR-146a knockdowned OSCC cells or miR-146a knockout OSCC mice model, are indispensable to further confirm the findings of this study.

## Conclusion

miR-146a was overexpressed in OSCC tissues and cell lines. miR-146a overexpression and knockdown in OSCC cell lines enhanced and inhibited cell migration/invasion, respectively. HTT was underexpressed in OSCC, and the expression pattern was inversely correlated with the miR-146a. The knockdown of HTT in OSCC cells enhanced cell migration/invasion. Higher HTT expression in cancer tissues was associated with longer survival percentage and duration. miR-146a knockout OSCC mice model not only showed higher expression of HTT in tongue tissue but also mitigates the OSCC progression and tongue epithelial layer damage. Our results suggest the involvement of the miR-146a/HTT axis on OSCC cell migration and invasion.

## Data Availability Statement

The original contributions presented in the study are included in the article/**Supplementary Material**. Further inquiries can be directed to the corresponding authors.

## Ethics Statement

The studies involving human participants were reviewed and approved by Ethical Institutional Review Board of Affiliated Stomatology Hospital of Guangzhou Medical University. The patients/participants provided their written informed consent to participate in this study. The animal study was reviewed and approved by Guangzhou Medical University Institutional Animal Care and Institutional Biosafety Committee.

## Author Contributions

LPW, YY, YC, JP, and LG designed the study, interpreted the data, and finalized the manuscript. YC, XG, YF, LJW, and YS contributed to perform experiment, data collection, and interpretation. All authors contributed to the article and approved the submitted version.

## Funding

This study was supported by the project of Guangzhou Science and Technology Bureau (2020020301), Department of Education of Guangdong Province (2018KTSCX186), Bureau of Technology Industry Business and Information of Liwan District, Guangzhou city (201804015), and The Project of Guangzhou Municipal Health Commission (20181A011103). High-level university construction funding of Guangzhou Medical University (02-412-B205002-1003018). The funding bodies played no role in the design of the study and collection, analysis, and interpretation of data and in writing the manuscript.

## Conflict of Interest

The authors declare that the research was conducted in the absence of any commercial or financial relationships that could be construed as a potential conflict of interest.

## Supplementary Material

Supplementary Figure 1 Scheme of OSCC development in miR-146a knockout mice.

Supplementary Figure 2 The knockdown of miR-146a inhibited the OSCC cell invasion in transwell assay. **(A)** Representative microscopic images of OSCC cell invasion. **(B)** Quantitative analysis of invasive cells. Data are presented as mean ± SD form three independent experiments in triplicate. Significant effect of the miR-146a inhibitor treatment, *P < 0.05, NC, negative control. Scale bar: 100 μm.
