# Supplementary figures and images for: miR-146a Overexpression in Oral Squamous Cell Carcinoma Potentiates Cancer Cell Migration and Invasion Possibly via Targeting HTT
Source: Front Oncol. 2020 Nov 13;10:585976. doi: 10.3389/fonc.2020.585976 (PMC7691531; doi:10.3389/fonc.2020.585976)

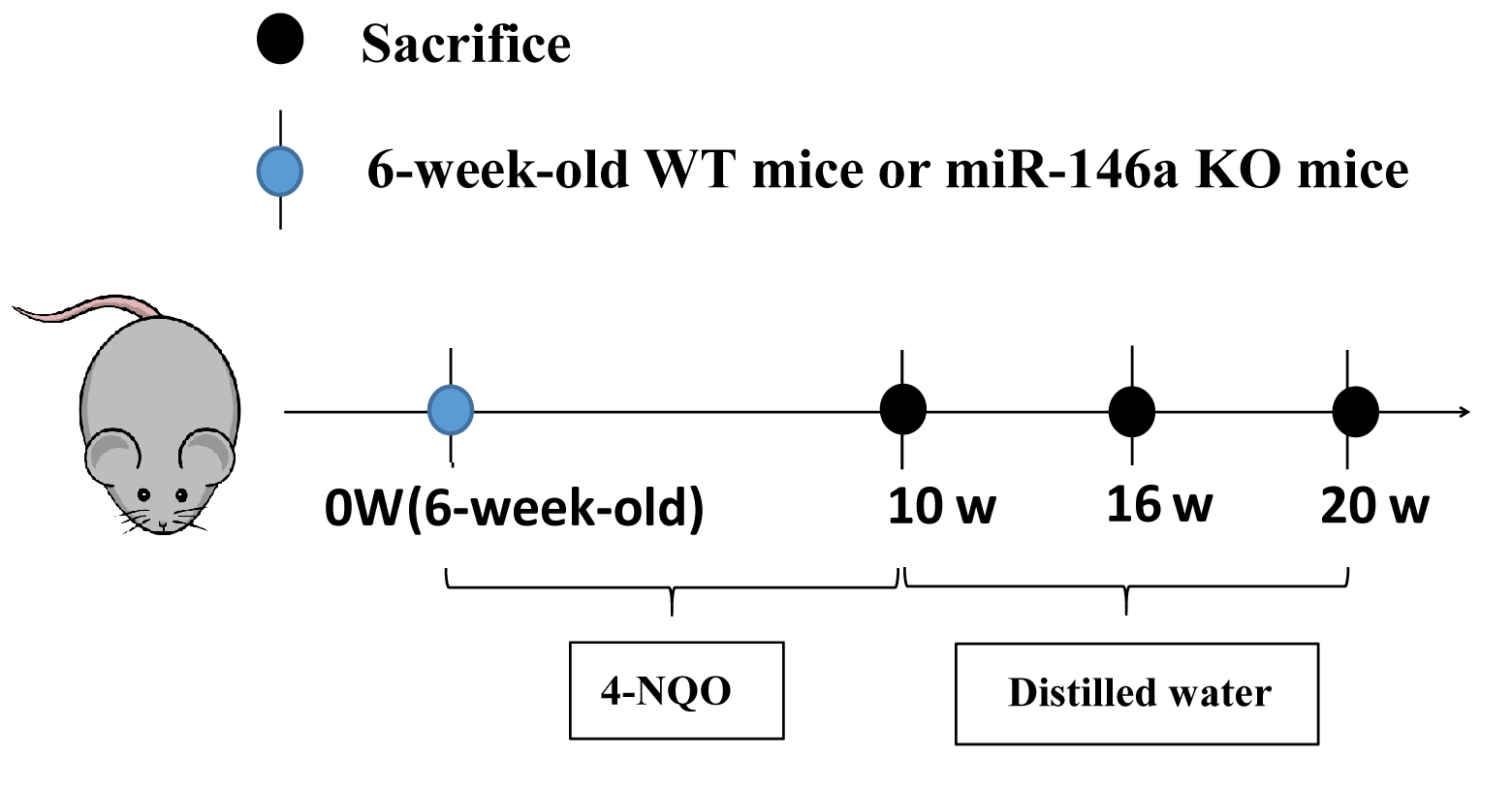

Supplement: Supplementary Figure 1 — Scheme of OSCC development in miR-146a knockout mice. [file Image_1.tif]

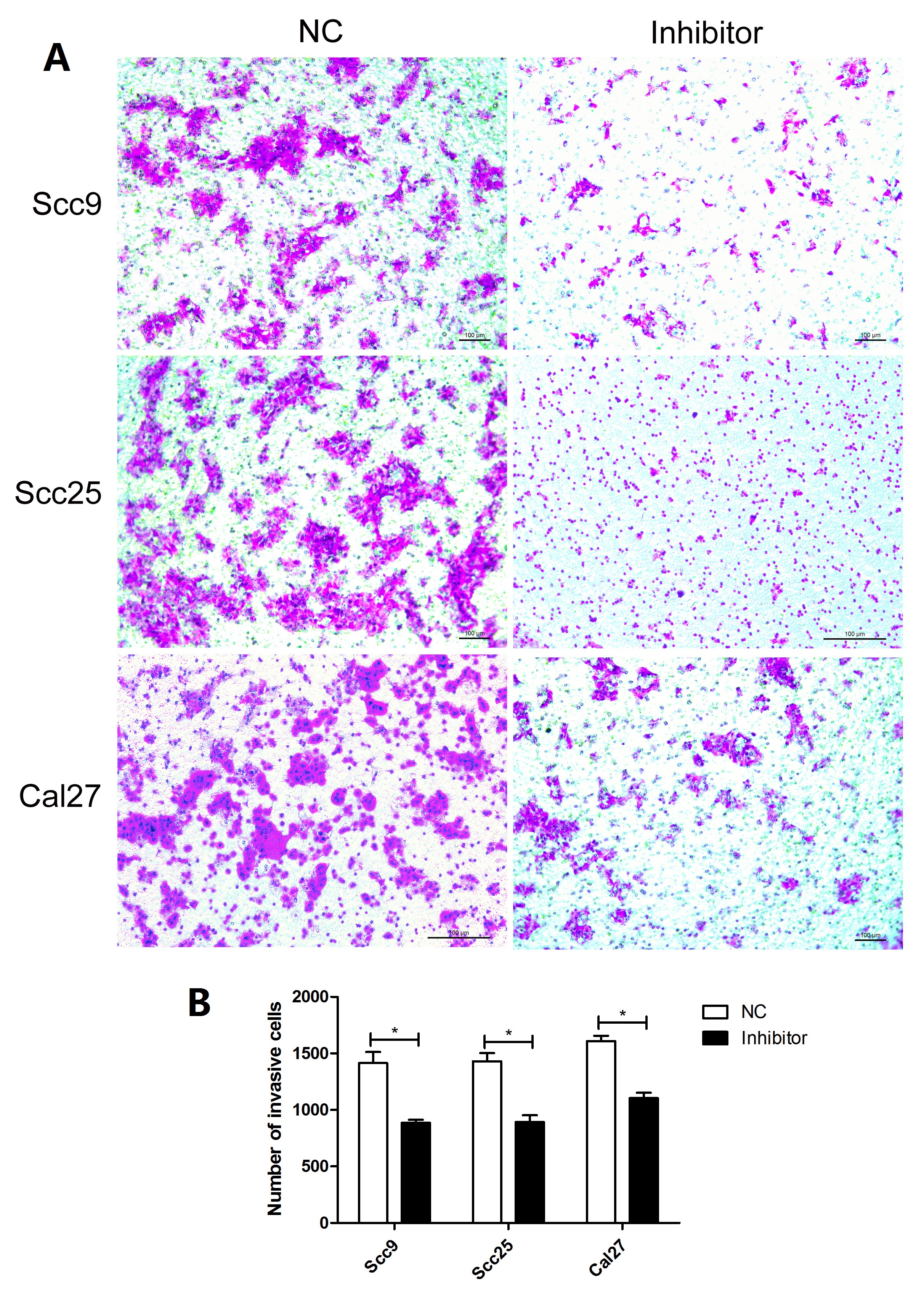

Supplement: Supplementary Figure 2 — The knockdown of miR-146a inhibited the OSCC cell invasion in transwell assay. (A) Representative microscopic images of OSCC cell invasion. (B) Quantitative analysis of invasive cells. Data are presented as mean ± SD form three independent experiments in triplicate. Significant effect of the miR-146a inhibitor treatment, *P < 0.05, NC, negative control. Scale bar: 100 μm. [file Image_2.jpeg]
